# One-way Acoustic Beam Splitter

**DOI:** 10.1038/s41598-018-29579-0

**Published:** 2018-09-11

**Authors:** Yifan Tang, Yifan Zhu, Bin Liang, Jing Yang, Jun Yang, Jianchun Cheng

**Affiliations:** 1 Key Laboratory of Modern Acoustics, MOE, Institute of Acoustics, Department of Physics, Collaborative Innovation Center of Advanced Microstructures, Nanjing University, Nanjing, 210093 P. R. China; 2 Key Laboratory of Noise and Vibration Research, Institute of Acoustics, Chinese Academy of Sciences, Beijing, 100190 P. R. China

## Abstract

As a key component of various acoustic systems, acoustic beam splitter (BS) finds important application in many scenarios, yet are conventionally based on the assumption that the acoustic waves propagate as easily when incident from either input or output side. It would therefore be intriguing, from the viewpoints of both science and technology, to break through this limit by realizing acoustic BSs supporting asymmetric transmission. Here we propose the concept of one-way acoustic BS capable of splitting acoustic beam incident from the input port into multiple beams while effectively reducing the backward transmission from any of the output ports. Furthermore, our design enables flexibly adjusting the number and angle of output beams by blocking the unused line defects. The numerical results verify the theoretical predictions and demonstrate the phenomenon of one-way acoustic BS at the predesigned frequency. Our design with functionality and flexibility bridges the gap between acoustic diodes and BSs and may enable novel multi-functional devices with great application prospects in diverse fields such as acoustic integrated circuits and acoustic communication.

## Introduction

Acoustic BS plays key role in design and fabrication of a great variety of acoustic systems, with broad application potentials in many scenarios ranging from acoustic information processing to biomedical diagnosis. In comparison with the conventional acoustic BS relying on natural materials with limited acoustical properties, the recent advent of artificial acoustic materials helps to improve the performance of acoustic BSs in terms of efficiency and flexibility, arousing considerable research interests during the past decade^1–8^. Li *et al*. demonstrate an acoustic BS by introducing a line defect structure, which allows for tuning the splitting efficiency by varying the radius of the rods in the PnC^9^. The line-defect-based mechanism for acoustic beam splitting in PnCs has been generalized to have multiple outputs by employing more line defects^10^. A defect-free beam splitting device is also proposed at the low GHz frequency regime, which provides a possible way to relax the limitations on the excitation source^11^. Acoustic BSs have also been designed on the basis of metamaterials that have extraordinary equivalent parameters such as negative mass density^12^ or zero index metamaterials^13^ and enable many fascinating wave manipulation effects^14–18^. However, the existing designs of acoustic BSs are proposed based on the basic assumption that the transmission of acoustic wave is symmetric along the two opposite directions, suggesting that the incident acoustic energy is allowed to pass through the whole system and be split into multiple beams regardless of the port the wave enters. The past few years witness a rapid expansion of the research field of asymmetric acoustic manipulation, leading to the emergence of the theoretical model^19^ and experimental prototype^20^ of an acoustic diode composed of a PnC and a strongly nonlinear medium and subsequent development of many PnC-based acoustic one-way devices with improvements in performance^21–23^. Considering the revolutionary scientific and technological significance that may be brought about by the breaking of transmission symmetry in an acoustic system, it can be expected that the realization of acoustic BSs with unconventional capability of supporting asymmetric transmission when splitting acoustic beams would offer new possibilities for acoustic manipulation and may enable novel artificial devices with versatile combined functionalities and broad application potentials.

In this Letter, we propose the concept of one-way acoustic BS capable of breaking through the above fundamental limit. This is enabled by a different mechanism that uses the combination of a line defects acting as waveguides in a PnC and a cavity defect with specially-designed asymmetric geometry to break the space-reversal symmetry and thereby support asymmetric cavity mode that couples to the waveguide modes with coupling strength quite different along opposite directions. As a consequence, the resulting device is able to split the acoustic energy incident from the only input port equally into multiple output ports with near-unity trasnsmission efficiency, while leading to effective reduction in the reversed transmission of acoustic waves coming from each of these output ports. In addition, the proposed one-way acoustic BS allows for flexibly adjusting the number and angle of output waves. We demonstrate the effectiveness of our proposed mechanism via numerical simulations, by inspecting the modes for the introduced cavity defect and line defect separately and then calculating the spatial pressure distribution for the transmitted wave in the combined system in the positive incidence and negative incidence cases.

## Results

### The introduction of one-way acoustic BS

**Figure 1:**
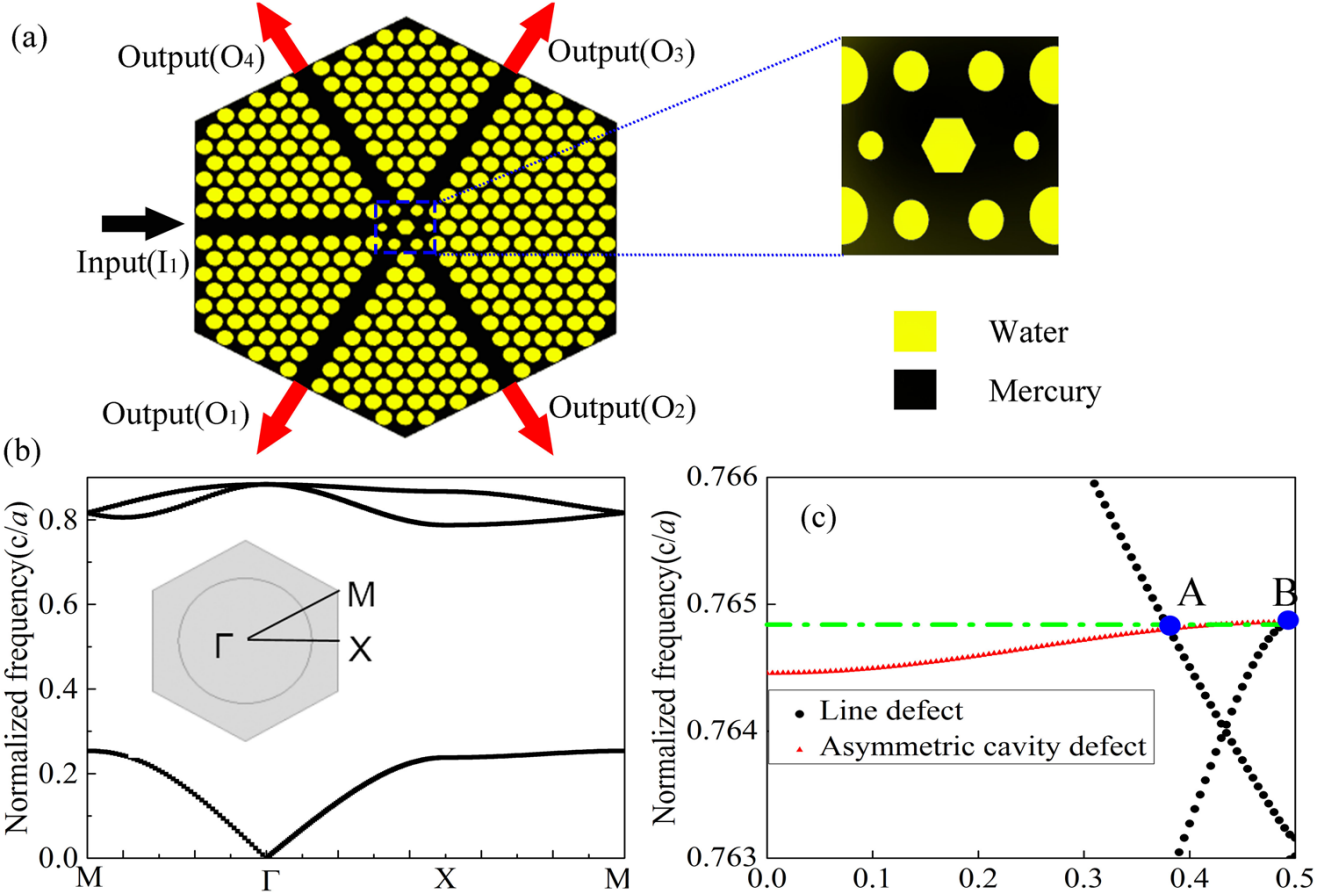
Schematic illustration and principle of generating of one-way acoustic BS. (**a**) Schematic of one-way acoustic BS. Inset: Zoom-in view of the asymmetric cavity defect consisting of the regular hexagon and two different types of scatterers. (**b**) Band structure of perfect PnC. (**c**) Band structure of the asymmetric cavity defect and line defect.

The schematic of our proposed one-way acoustic BS is illustrated in Fig. 1(a), which is formed by introducing defects with broken symmetry into a PnC which consists of the asymmetric cavity defect and the line defect. In the current design, we consider a 2D system and use a regular hexagon structure with five ports including one input port (marked by black arrow) and four output ports (marked by red arrows with the horizontal angle of ±60° and ±120° respectively). In our design, the positive incidence (PI) and negative incidence (NI) refer to the cases in which the acoustic wave incident from the input port side and output port side respectively. For breaking the symmetry in the geometry, the line defect is realized by removing a row of water scatterers, and the asymmetric cavity defect is formed by replacing the central scatterer by a regular hexagonal one whose side length is 0.389*a* being the lattice constant and changing the sizes and locations of the six surrounding cylinders. To be specific, around the regular hexagon we use two kinds of scatterers whose radii are chosen as 0.5*a* and 0.7*a* respectively, each of which is displaced by 0.2*a* away from the center. Figure 1(b) shows the band structure of the perfect PnC in the first Brillouin zone. For simplicity without losing generality, the perfect PnC is composed of water cylinders arranged in a triangular lattice and immersed in mercury, and the radius of each cylinder is chosen as $0.36a$. It is clear that there is a large bandgap ranging from $0.241c/a$ to $0.793c/a$ along the ΓΧ direction, which is convenient to choose the operating frequency for the line defects and asymmetric cavity defect^24^.

### The mechanism of one-way acoustic BS

For clarifying the fundamental mechanism underlying our design, we give analysis to the wave propagation processes in the two major components of the proposed model - the cavity defect and line defect - individually. The dispersion curves of the cavity defect and line defect are calculated and plotted in Fig. 1(c). It is seen that the two kinds of curves representing the line and asymmetric cavity defect modes (marked by black dots and red triangles respectively) intersect with each other at the frequency of $0.7648c/a$ in two specific points, denoted as A and B respectively. Figure 1(c) shows that at point A, there simultaneously occur two different modes for wave vector ${k}_{1}$ at $0.38(2\pi /a)$ in the PnC, i.e., the line mode and asymmetrical cavity mode. Similarly, the point B also contains two modes, which are the line and asymmetrical cavity modes for wave vector ${k}_{2}$ at $0.5(2\pi /a)$. In other words, by tuning the structural parameters appropriately one can produce identical cavity modes and different line modes at points A and B that correspond to one frequency, making it possible to achieve asymmetric coupling between these modes as the wave vector varies.

**Figure 2:**
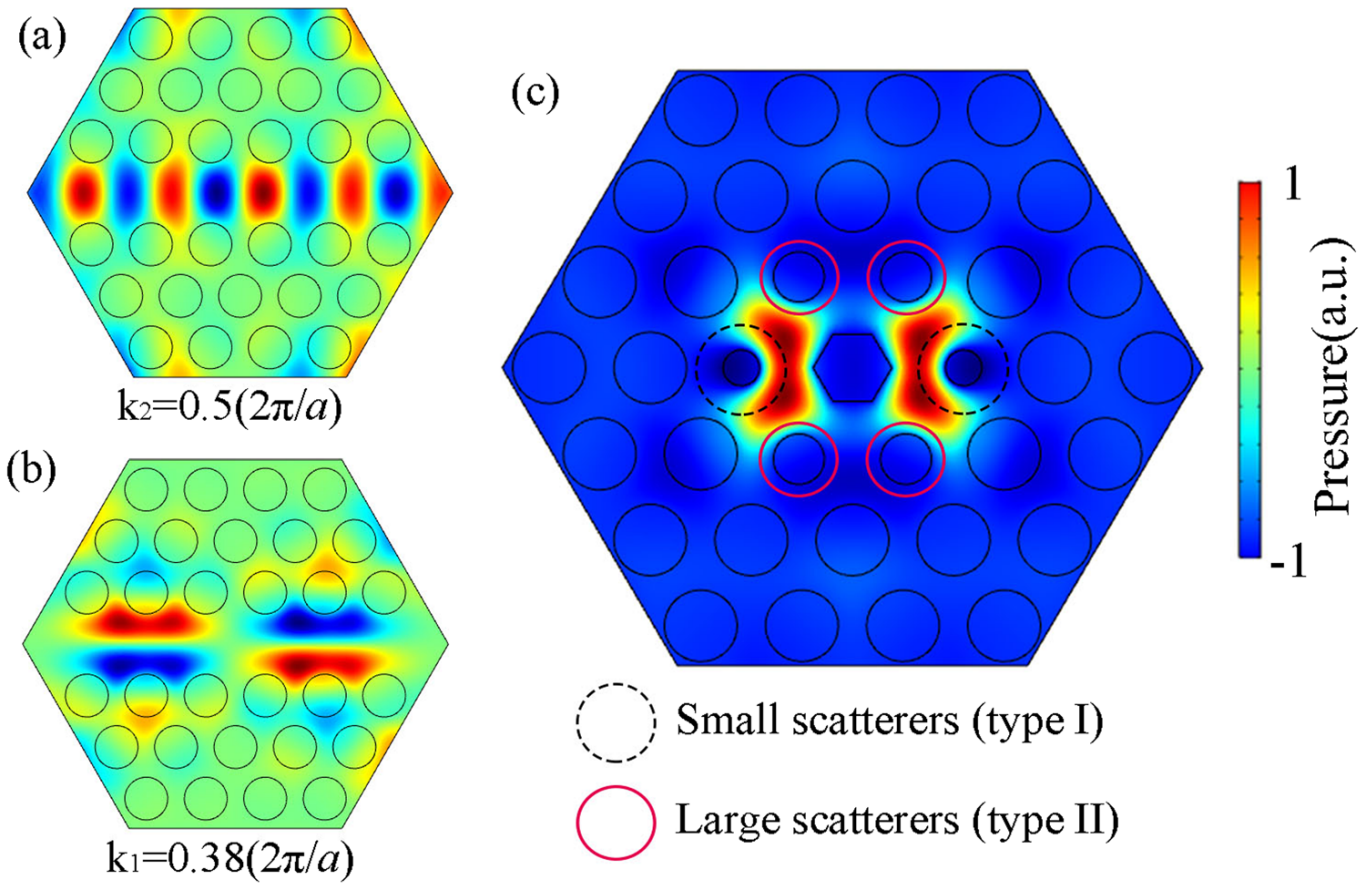
Different eigenfield patterns in line and cavity defects. The propagation modes of the waveguide for (**a**) 0^th^-order mode; (**b**) 1^st^-order mode. (**c**) The eigenfield pattern of the asymmetrical cavity defect.

In our model, the line defect (formed by removing a column of water scatterers in the PnC) is for guiding the incident acoustic wave to propagate within it. Figure 2 illustrates the spatial distribution of acoustic pressure in a supercell structure at a particular frequency of $f=0.7648c/a$. We calculate the acoustic propagation in such a waveguide system via numerical simulations and plot the typical results in Fig. 2(a,b) which prove that at this specific frequency there are two modes with different wave vectors as predicted by the dispersion relationship given by Fig. 1. The numerical results clearly show that the acoustic field pattern for ${k}_{2}$ at $0.5(2\pi /a)$ only allows the lowest 0^th^-order mode to propagate, which is symmetric with the propagation axis. In contrast, the spatial distribution of acoustic pressure in the 1^st^-order mode is antisymmetric with respect to the propagation axis for ${k}_{1}$ at $0.38(2\pi /a)$.

The aforesaid waveguides used for conducting the propagating modes are connected together by a cavity defect with asymmetric geometry, which will be demonstrated in what follows to play a vital role in the production of the desired asymmetric transmission in the resulting BS. Next, we consider the acoustic propagation in a supercell containing asymmetric cavity defect and plot the eigenfield pattern at the same frequency with line defects, as schematically shown in Fig. 2(c). The small and large scatterers in the dashed circles and circles are denoted as “I” and “II”, respectively. The numerical results clearly show a cavity mode with asymmetric spatial pattern, as manifested by the azimuthally-dependent spatial distribution of acoustic energy resulting from the standing waves between the central hexagonal cylinder and the neighboring ones. Therefore, the scatterer I in the asymmetric cavity mode can couple with 0^th^-order mode with high efficiency, because the neighboring pressure of the scatterer I is symmetrical with the propagation axis. Similarly, the scatterer II in the asymmetric cavity mode can couple to 1^st^-order mode, as the nearby pressure is antisymmetric to the output axis. This suggests the possibility of producing asymmetric transmission if we introduce these two kinds of defects (viz., cavity and line defects) into the PnC simultaneously, which gives the one-way BS illustrated in Fig. 1(a). It can be expected that, in such a hybrid structure comprising these two key components, the asymmetric cavity mode will couple to different waveguide modes supported by each line defect with quite different strength. Specifically, there will exist strong coupling between the cavity mode and the 0^th^-order mode on the joint between the input waveguide and central cavity (near scatterer I) due to the fact that the acoustic pressure distribution of the cavity mode on the scatterer I is symmetric with the propagation axis and consistent with that of the lowest waveguide mode demonstrated in Fig. 2(a). Similarly, the coupling strength between the 1^st^-order mode and cavity mode on the joint near scatterer II will also be strong because of their uniform spatial patterns with both being antisymmetric to the propagation axis. In contrast, for incident plane waves that propagates as the lowest 0^th^-order mode in each line defect connected to scatterer II, the incident acoustic energy will not couple effectively with the cavity mode owing to the discrepancy between the spatial patterns of the cavity mode and waveguide mode. This extraordinary effect is based on a mechanism that uses phononic crystals (PnCs) containing multiple line defects connected by one specially-designed cavity defect to support coupling between the lowest and higher waveguide modes which is high efficiency for positive incidence case but low efficiency as the incident direction is reversed. As a consequence, this mechanism could enable a high efficiency transmission for acoustic wave incident from the input port but substantially suppress the propagation as the wave comes from any of the output ports.

### Acoustic waves transmission in PI and NI cases with different angles

**Figure 3:**
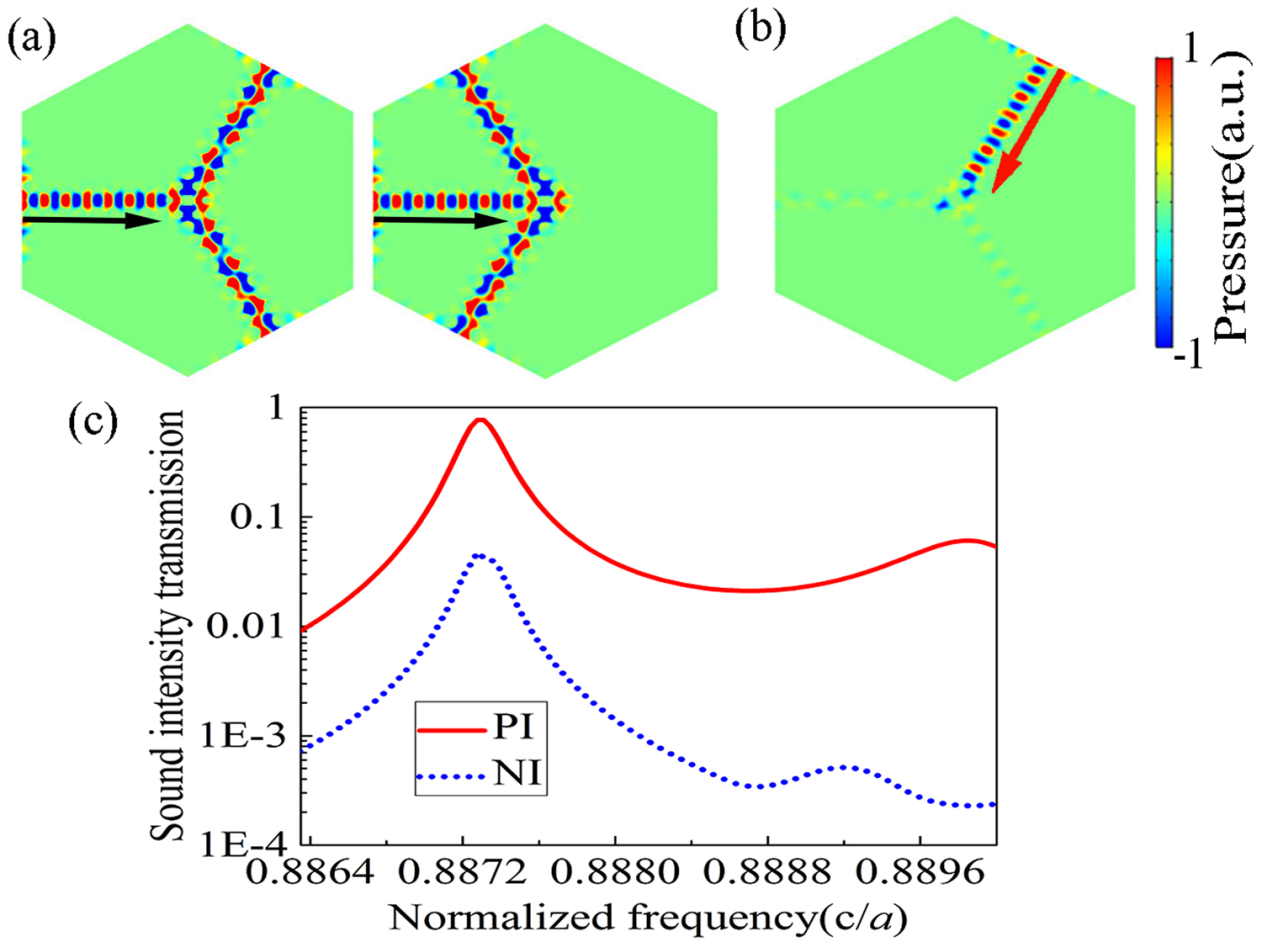
Results of one-way BS with different output angles. (**a**) The spatial distributions of acoustic pressures in the PI case for one-way BSs formed by blocking the upper-left output passage O_4_ and bottom-left passage O_1_ (Left subfigure), and by blocking the upper-right passage O_3_ and bottom-right passage O_2_ (Right subfigure). (**b**) The pressure field distributions of one-way BS in NI case. (**c**) Transmission curves with the PI (red line) and NI (blue dashed line) cases.

We verify the effectiveness of the proposed mechanism via numerical simulations by calculating the transmission property for the incident acoustic waves propagating along different angles in the designed one-way acoustic BS device comprising the aforementioned two major components. As typically demonstrated in Fig. 3(a), one-way acoustic BS with two outputs can be realized by simply obstructing the other two untapped exit ports, allowing the outgoing waves to leave along two horizontal angles that are ±60° or ±120°. For PI case, with the uniform spatial patterns of the waveguide and asymmetric cavity, the 0^th^-order mode can couple to the asymmetric cavity mode near the scatterer I and the 1^st^-order mode can also couple with this cavity mode near the scatterer II. Therefore, the 0^th^-order mode is converted into the 1^st^-order mode in the exit ports so that acoustic waves can be transmitted efficiently as expected. Figure 3(b) shows the spatial pattern of NI case which is the 0^th^-order mode incident from the Output (O_3_) and proves that there is a discrepancy between the asymmetrical cavity mode and the 0^th^-order mode near the scatterer II. Figure 3(c) illustrates the sound intensity transmission property of the designed one-way acoustic BS along two opposite directions. It can be observed that for the PI case the maximal sound inensity transmission almost reaches unity at the target frequency of $0.8873c/a$ where the value for the NI case is about 0.05. The peak value of total transmission of PI case is near two orders of magnitude larger than that of NI case. It is seen that the operating frequency of numerical results for our designed model of one-way acoustic BS is slightly different with the common frequency calculated respectively of waveguide modes and asymmetric cavity mode on account of the finite size and unavoidable coupling between the asymmetric cavity defect and line defects^25,26^.

### Acoustic waves propagation with different numbers of outputs

**Figure 4:**
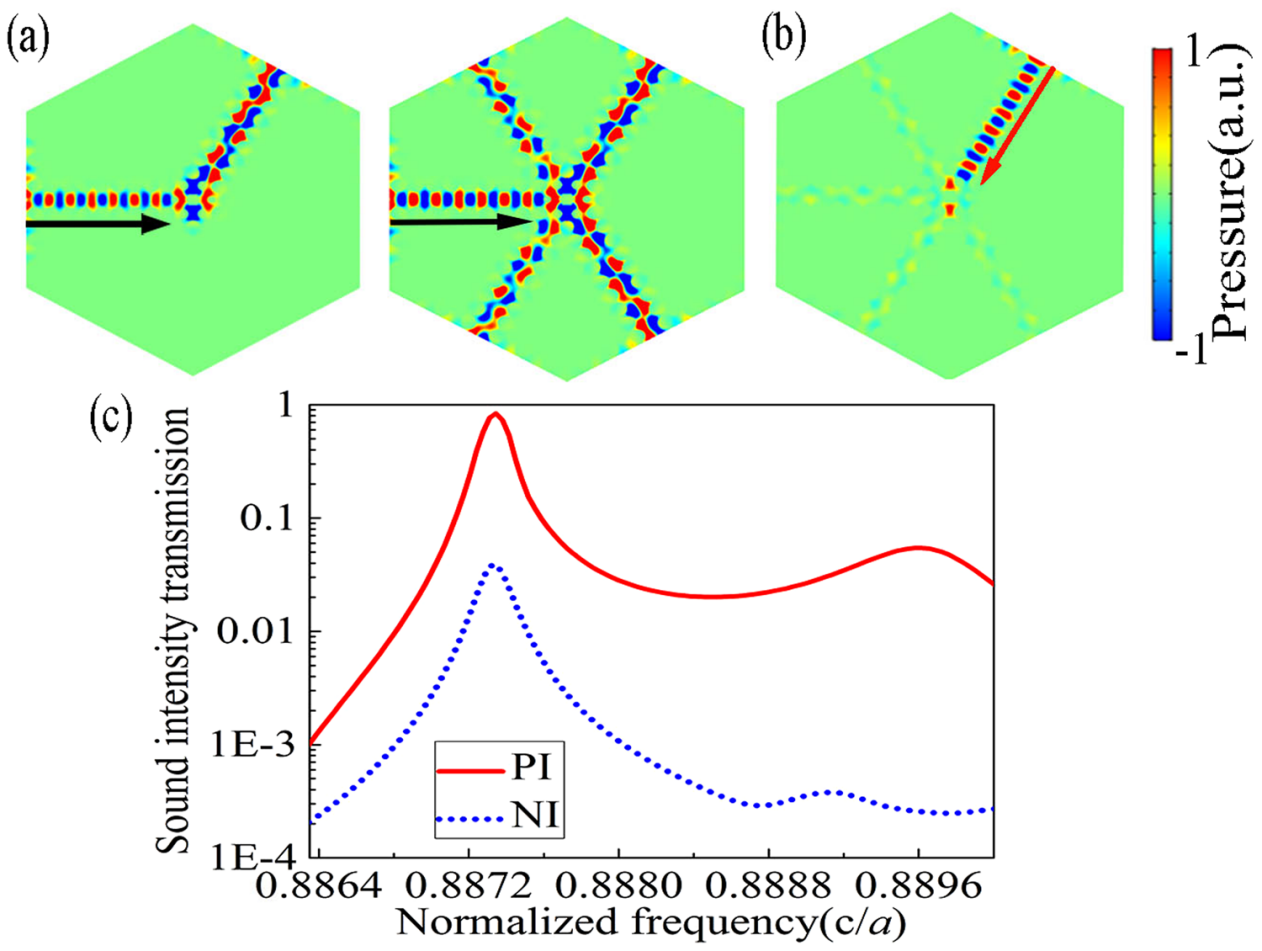
Results of one-way BS with different numbers of outputs. (**a**) The spatial distributions of acoustic pressures in the PI case for one-way BS formed by blocking all the output passages except for the upper-right one O_3_ (Left subfigure), and by leaving all the output passages unblocked (Right subfigure). (**b**) The pressure field distributions of one-way BS in NI case. (**c**) Frequency dependence of transmission curves for PI and NI cases.

Next, we demonstrate the flexibility of our design in adjusting the number of outputs. Figure 4(a) shows the calculated pressure field distributions for one-way acoustic BS with one and four outputs, which is for PI case. As manifested by the numerical results, the 0^th^-order mode is converted into the 1^st^-order mode quite strongly in our model with different outputs due to their different structural parameters. As for NI case where the wave incident from the output port O_3_ in an one-way acoustic BS with four outputs as depicted in Fig. 4(b), the acoustic field patterns of the 0^th^-order mode and the asymmetric cavity mode cannot couple with each other near the scatterer II. We calculate the total transmissions for both the PI and NI cases and plot the numerical results in Fig. 4(c). The realization of one-way acoustic BS, as evidence by the asymmetric transmission curves for two opposite directions, proves that the effectiveness of our proposed scheme for breaking the transmission symmetry in common beam-splitting effect, which may be enlightening for the further development of novel functional devices and enable more versatile wave-steering phenomena with wide applications.

## Discussion

In summary, we have presented the concept of one-way acoustic BS that breaks through the limit in previous BS designs supporting only symmetric acoustic transmission and offers the possibility of splitting incident acoustic wave into multiple beams while reducing the transmission of backward waves. For the proposed PnC-based acoustic BS design composed of line defect and cavity defect, we elucidate the underlying mechanism that controls the transmission of asymmetric cavity defect to achieve the one-way BS. Furthermore, our design idea allows for flexibly adjusting the number and angle of outputs in the one-way acoustic BS. The theoretical prediction is verified by numerical simulations, and the performance of the resulting device is demonstrated via the production of one-way beam splitting effect. It is worth stressing that our proposed mechanism is general and also applies to PnCs compose of solid and liquid. Considering the presence of complex modes caused by mode conversions on solid-liquid interfaces and the lowered impedance contrast between solids and liquids, however, high-efficiency one-way performance is more difficult to achieve in solid-liquid composites, which needs a complicated optimization design of structural parametres. The next step of our work is to achieve broad and tunable operating frequency such as by using active-control techniques and to design one-way BS in solid-liquid system due to the simplicity of its experimental realization. Our design with capability and flexibility constitutes a major step towards integration of the functionalities of acoustic one-way devices and BSs that are conventionally perceived irrelevant and opens up possibilities for the design and application of multi-functional acoustic devices, which would may have significant and far-reaching impact on many diverse fields such as acoustic communication and biomedical diagnosis.

## Methods

Throughout the paper, the numerical simulations are performed by the finite element method based on commercial software COMSOL Multiphysics. The materials applied in the simulations are water and mercury. The density and sound speed are ${\rho }_{{\rm{w}}}=1000\,{\mathrm{kg}/{\rm{m}}}^{{\rm{3}}}$ and ${c}_{{\rm{w}}}=1490\,{\rm{m}}/{\rm{s}}$ for water, and $\rho =13500\,{\mathrm{kg}/{\rm{m}}}^{{\rm{3}}}$ and $c=1450\,{\rm{m}}/{\rm{s}}$ for mercury, respectively. For Figs 1(b) and 2, the periodic boundary condition are employed in the boundaries to calculate the Band structure of perfect PnC and the eigenfield patterns. Plane wave radiation boundary condition is imposed on the incident boundaries to simulate spatial distribution of acoustic pressures,as shown in Figs 3 and 4. Considering the plane wave radiation boundaries are not sufficient to absorb the oblique incidence waves, the perfectly matched layers are used to eliminate the reflected waves by the outer boundaries.
